# Ischemic volvulus of the transverse colon: A case report and review of literature

**DOI:** 10.1186/1757-1626-1-174

**Published:** 2008-09-22

**Authors:** Dorothy A Sparks, Murtaza Y Dawood, Daniel M Chase, David J Thomas

**Affiliations:** 1 Northside Medical Center/Northeast Ohio Universities College of Medicine, Department of Surgery, 500 Gypsy Lane, Youngstown, OH 44505, USA

## Abstract

A 75-year old male presented to the emergency room with worsening abdominal pain and distension. Plain radiographs were suggestive of a large bowel obstruction due to volvulus. An attempt to detorse the volvulus and decompress the colon endoscopically failed, after which the patient was taken for an exploratory laparotomy. A transverse colon volvulus was found, and an extended right hemicolectomy and ileostomy was performed. We discuss the diagnosis and management of transverse colon volvulus and review the pertinent literature.

## Case presentation

A 75-year old Caucasian male presented to the emergency department with severe abdominal distension and mild dyspnea. A retired auto factory worker, he was 5 feet 9 inches tall and 250 pounds. His medical history was significant for diabetes mellitus, coronary artery disease, and a myocardial infarction. He had undergone bilateral inguinal hernia repairs. Medications included metoprolol, aspirin, enalapril, and metformin. The patient was reformed smoker, and drank alcohol occasionally. His family history was significant for diabetes. The patient also admitted with a history of chronic constipation for which he often self-medicated with magnesium citrate. Prior to presentation his efforts had been unsuccessful, and he had not had a bowel movement for ten days.

Physical examination revealed a thin elderly gentleman with a massively distended abdomen; it was tympanic to percussion with minimal bowel sounds and visibly dilated loops of bowel. The abdomen was non-tender and there were no signs of peritonitis. Digital rectal examination revealed an empty rectal vault and no intraluminal masses.

**Figure 1:**
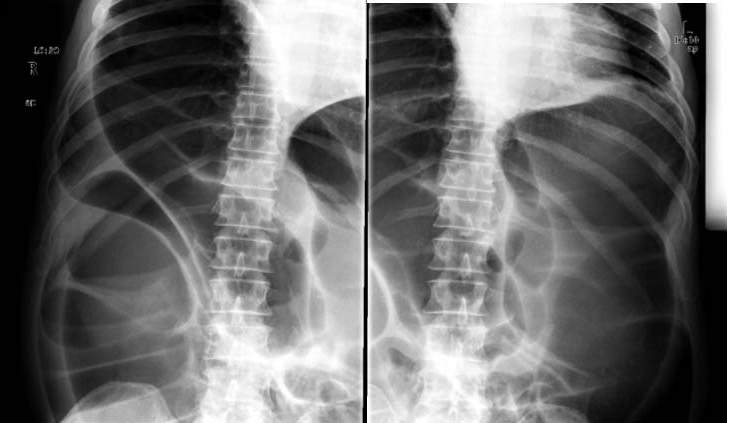
**Radiographs demonstrating massively dilated large bowel.** The transverse colon had a maximum diameter of 18 cm.

**Figure 2:**
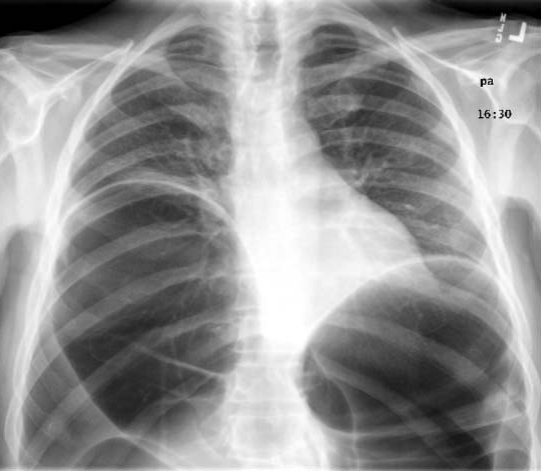
Admission chest radiograph demonstrating elevation of both hemidiaphragms.

Laboratory studies demonstrated leukocytosis, hypokalemia, and prerenal azotemia. Abdominal radiographs were obtained and showed massively distended large bowel, and was suggestive of obstruction due to volvulus (Figure 1). Both hemidiaphragms were elevated because of the dilated large bowel, which likely explained the patient's dyspnea (Figure 2).

Due to the massive distension, we decided against performing a barium enema. Instead, we elected to attempt endoscopic decompression and detorsion. This was preformed in the operating room to allow for the possibility of failure with conversion to laparotomy. We were unable to advance the endoscope beyond the splenic flexure. The left colon appeared to be decompressed, but massive distension involving the transverse and ascending colon persisted. These findings were confirmed with an abdominal radiograph. The patient was then prepared for laparotomy.

**Figure 3:**
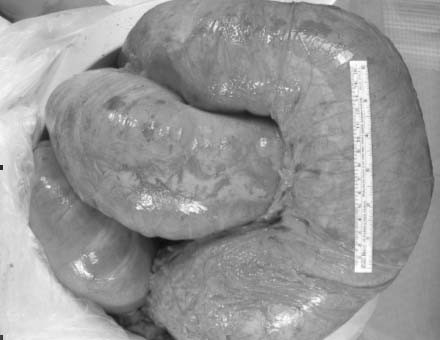
Gross specimen post-colectomy.

Intraoperative findings demonstrated a rotation of the transverse colon on its mesentery, causing a closed loop obstruction. The volvulus was delivered into the incision and detorsed (Figure 3). The entire colon – from the cecum to the sigmoid colon – appeared massively dilated. We performed an extended right hemicolectomy with a Brooke ileostomy. The patient made a satisfactory recovery and was discharged from the hospital six days later.

## Discussion

The incidence of transverse colon volvulus is relatively rare when compared to cecal volvulus and sigmoid volvulus. The term volvulus, derived from the Latin word *volvere *meaning "to turn," [1] describes the abnormal twisting of the bowel often leading to a closed-loop obstruction. This twisting usually occurs along the mesenteric axis of the bowel, resulting in venous obstruction and eventually arterial compromise[2]. Transverse colon volvulus was first categorized by Eisenstat *et al *as either acute fulminating or subacute progressive [2-5].

Acute fulminating transverse colonic volvulus is usually described in the setting of marked leukocytosis, acute abdominal pain with rebound tenderness, nausea and vomiting, but limited abdominal distension [3]. Bowel sounds on initial presentation are absent or very quickly become so [2]. Immediate surgical intervention is required in order to resect compromised bowel before gangrene and perforation occurs [5].

Subacute progressive transverse volvulus is associated with massive abdominal distension in the setting of mild abdominal pain without rebound tenderness and little or no nausea or vomiting [3]. The leukocyte count is often normal or only mildly elevated, attributable to the lack of ischemia at early stages [2]. Failure to provide timely treatment at this subacute stage often results in the disease progressing to the acute fulminating type [5].

The two properties essential to the formation of a volvulus are redundancy and non-fixation [2,6]. The ascending and descending segments of the colon are fixed, but the sigmoid colon, cecum, and transverse colon are mobile within the peritoneum, tethered by their mesentery [2,6,7]. This mobility allows volvulus to occur at these locations. Redundancy of any of these segments further enables the formation of a volvulus [6].

The most common colonic volvulus is in the sigmoid colon [8]. It often occurs in the elderly or in psychiatric patients, and accounts for approximately 8% of all intestinal obstructions [9]. The initial treatment is sigmoidoscopy with decompression and detorsion [2]. A sigmoid colectomy is recommended during the same hospitalization due to a high recurrence rate [2,10].

Cecal volvulus occurs at the level of the ascending colon. The mobility of the cecum is attributed to a defect in peritoneal fixation [11]. Similar to a sigmoid volvulus, a cecal volvulus is treated with endoscopic decompression followed by surgical resection. However, cecal volvulus carries a higher mortality rate [12], and endoscopy is less successful in treating it [8].

Transverse colon volvuli are extremely uncommon. It was first reported in 1932 as a cause of colonic obstruction by the Finnish surgeon Kallio [13]. To date, the surgical literature has reported fewer than 100 cases worldwide, most of which are discovered at the time of surgery [5]. A transverse colon volvulus does not have the same classically recognizable radiographic features as sigmoid and cecal volvuluses. Some authors, looking retrospectively, have suggested that its appearance can be recognized on radiographs as "loops of dilated large bowel with two air fluid levels [3]." The gold standard of diagnosis is a contrast enhanced plain film which reveals the "birds beak" phenomenon characteristic of any volvulus.

Regrettably, it is difficult to draw any but the most broad conclusions regarding the occurrence of transverse colon volvulus in the adult population due to the relatively small number of reported cases. Though generally associated with congenital malrotation [14], an association has been reported with Chilaiditis syndrome [5,6], *Clostridium difficile *pseudomembranous colitis [2], and impaired intestinal motility associated with pregnancy [15].

## Conclusion

All forms of colonic volvulus clinically present as a large bowel obstruction. Though rare, the possibility of a transverse colon volvulus must always be part of a differential diagnosis when dealing with this clinical situation.

## Consent

Written informed consent was obtained from the patient for publication of this case report and accompanying images. A copy of the written consent is available for review by the Editor-in-Chief of this journal.

## Competing interests

The authors declare that they have no competing interests.

## Authors' contributions

DS wrote the presentation of the case, and contributed to the discussion section. MD researched the literature review on colon volvulus and contributed to the discussion section. DC edited and revised the entire manuscript and was a major contributor to the discussion section. DT researched the patient information as well as the figures, and was a major contributor to the presentation section of the manuscript. All authors read and approved the final manuscript.
